# Retention of medical officers in district health services, South Africa: a descriptive survey

**DOI:** 10.3399/BJGPO.2022.0047

**Published:** 2022-11-02

**Authors:** Robert Mash, Beverley Williams, Dusica Stapar, Gavin Hendricks, Herma Steyn, Johann Schoevers, Leigh Wagner, Mumtaz Abbas, Paul Kapp, Stefanie Perold, Steve Swartz, Werner Viljoen, Muideen Bello

**Affiliations:** 1 Family Medicine and Primary Care, Stellenbosch University, Cape Town, Western Cape, South Africa; 2 Rural Health Services, Western Cape Government, Cape Town, South Africa; 3 Metro Health Services, Western Cape Government, Cape Town, South Africa

**Keywords:** physicians, general practitioners, public sector, district health services, health workforce, retention

## Abstract

**Background:**

The health workforce is critical to strengthening district health services (DHS). In the public sector of South Africa, medical officers (MOs) are essential to delivering services in primary health care (PHC) and district hospitals. Family physicians, responsible for clinical governance, identified their retention as a key issue.

**Aim:**

To evaluate factors that influence retention of MOs in public sector DHS.

**Design & setting:**

A descriptive survey of MOs working in DHS, Western Cape, South Africa.

**Method:**

All 125 MOs working in facilities associated with the Stellenbosch University Family Physician Research Network (SUFPREN) were included in the survey. A questionnaire measured the prevalence of key factors that might be associated with retention (staying >4 years) and included the Satisfaction of Employees in Health Care (SEHC) tool and Short Warwick–Edinburgh Mental Wellbeing Scale (SWEMWBS). Data were collected in Research Electronic Data Capture (REDCap) and analysed in the Statistical Package for Social Sciences (SPSS).

**Results:**

Ninety-five MOs completed the survey. The overall rating of the facility (*P* = 0.001), age (*P* = 0.004), seniority (*P* = 0.015), career plans (*P*<0.001), and intention to stay in the public sector (*P*<0.001) were associated with retention. More personal factors such as social support (*P* = 0.007), educational opportunities for children (*P* = 0.002), and staying with one’s partner (*P* = 0.036) were also associated with retention. Sex, rural versus urban location, district hospital versus primary care facility, overtime, remuneration, and additional rural allowance were not associated with retention.

**Conclusion:**

The overall rating of the facility was important and subsequent qualitative work has explored the underlying issues. These findings can guide strategies in the Western Cape and similar settings to retain MOs in the DHS.

## How this fits in

To deliver effective DHS attention must focus on the health workforce. That includes the number, distribution, and competence of healthcare workers. International research has identified a variety of factors known to influence the retention of doctors in DHS. Family physicians in the Western Cape of South Africa identified retention of doctors in the public sector of DHS as a key issue. This study shows that doctors' overall experience of the facility as a place to work, social support, educational opportunities for children, and being able to live with their partner were all associated with retention.

## Introduction

There is a renewed global effort to strengthen PHC and primary (district) hospitals in order to achieve sustainable development goals and universal health coverage.^1,2^ The World Health Organization recently published an operational plan for implementing PHC^1^ and a new framework for measurement of performance.^3^

The framework recognises the importance of the workforce in strengthening DHS.^3^ The number, competency, and distribution of motivated healthcare workers are all important. Effective management, supervision, and appropriate remuneration of the healthcare workers has been emphasised. The framework also specifically mentions the relevance of family physicians and the availability of community health workers as particularly important to strengthen services.

In South Africa, non-specialist doctors working in the public sector are called ‘medical officers’ (MOs) and are essential members of the multidisciplinary teams in PHC and district hospitals. MOs are usually generalists in order to provide comprehensive care, but in large district hospitals may develop a special interest and even complete a postgraduate diploma in that area of interest. The teams that they work in typically include community health workers, clinical nurse practitioners, pharmacists, family physicians, other nurses, and allied health professionals. A recent South African report estimated that there will be a shortfall of 2293 MOs in PHC by 2025 and that inequitable distribution of MOs between provinces is an ongoing issue.^4^

In South Africa, the Ideal Clinic initiative defines norms and standards for PHC and recommends that every clinic has access to a primary care doctor.^5^ District hospitals continue to have skills gaps that can be addressed by competent MOs.^6,7^ Many of the available MOs, however, are working under obligation as interns or community service MOs, and are also inexperienced. Retaining permanent MOs, who grow in competence and can ensure the comprehensiveness and quality of services, is therefore important. This is particularly challenging in remote and rural areas.^8^ Specialising as a family physician, through 4 years of postgraduate training to specifically work in the DHS, is now also a career pathway and a strategy to grow and retain expertise.^9^ A new national Diploma in Family Medicine has also been introduced and should influence postgraduate training and promotion of MOs in the future.^10^

Many factors have been identified that influence the retention of MOs, and much of the literature is concerned with distribution to rural areas.^11–16^ Professional factors include adequate remuneration, appropriate workload, opportunities for professional development and career progression, adequate infrastructure and resources, effective management and support from specialists, and the ability to refer patients. More personal factors include flexibility of services to accommodate parenting, safety, recognition, educational opportunities for children, and career opportunities for one’s partner. Acceptable living and working conditions may also be important in poorly developed areas.

In the Western Cape a vacancy rate of 6% was reported for the DHS in 2020–2021, although no specific rate was given for MOs.^17^ The turnover of MOs and ability to retain individuals in the long term is also not reflected in such a vacancy rate. Family physicians are responsible for clinical governance in district hospitals and PHC,^18^ and identified the retention of MOs as a key issue impacting their ability to improve the quality and comprehensiveness of care. Having sufficient MOs, and a mix of senior and junior doctors, impacts on the ability to cover overtime rosters, run operating theatres, provide training for the whole multidisciplinary team, support PHC, and give attention to quality improvement activities.

The Stellenbosch University Family Physician Research Network (SUFPREN)^19^ prioritised a research question to investigate factors influencing the retention of MOs in the DHS. While a menu of factors that can influence retention has been identified, the factors that are important in a specific model of care and health service context must still be determined. Therefore, the aim was to evaluate the factors that influence the retention of MOs in public sector DHS in the Western Cape, South Africa.

## Method

### Study design

This was a descriptive observational cross-sectional survey by means of a questionnaire. The factors identified in this study will be further explored in a descriptive exploratory qualitative study,^20^ not reported here.

### Setting

In South Africa about 80% of the population are dependent on the public sector health services. This population is uninsured and from low socioeconomic groups, while the more affluent insured population utilise the private sector.

It is a district-based health system with 55 districts and metropolitan areas across nine provinces. The Western Cape Province includes one metropolitan area (Cape Town) and five rural districts, and has a population of approximately 7 million people. The population using the public sector are often poor, rural farmworkers or urbanised workers, living in informal settlements or basic housing. Historic racial inequities persist and most patients come from so-called African and Coloured groups, and speak English, Afrikaans, or isiXhosa.

The DHS includes both district hospitals and primary care facilities. Primary care facilities can be community health centres, which are open 24 hours a day and often include an emergency centre and midwifery obstetric unit; a community day centre, open during office hours; and clinics, which are smaller fixed or mobile facilities. District hospitals rely on MOs without any specialist training. Community health and day centres usually have a multidisciplinary team that includes MOs, while clinics are usually staffed by nurses. Nurse practitioners are the main primary care provider and first contact. There are also teams of community health workers in the catchment area and there is a commitment to a community-oriented primary care approach.

Many MOs are temporary as they are deployed to district health facilities as part of their 2-year internship or 1-year compulsory community service. Following this, there is a smaller cadre of permanent MOs who are gradually promoted from grade 1 to grade 3 with each 5 years of service. Permanent MOs, therefore, are the backbone of clinical care in the DHS.

The Western Cape has also committed to the employment of family physicians (specialists in family medicine) at district hospitals and community health centres as well as some community day centres. Family physicians have 4 years of postgraduate training as clinicians, capacity builders, and leaders of clinical governance.

### Study population and sampling

The study was conducted in facilities where the family physician had joined the SUFPREN. The network included seven community health centres and nine district hospitals on the service platform across all districts in the Western Cape. All 125 MOs working at these facilities were eligible for the study, whether full-time or part-time employed. MOs who were temporary were excluded, such as locums, interns, and community service MOs. Family physicians and registrars were also excluded. There was no sampling of the study population and all eligible MOs were invited, with a desired response rate of 80%.

### Data collection

The study was conducted between January and March 2021. Factors associated with retention were categorised into personal, professional, social, and financial.^11^ Questions were designed to measure the factors identified in the literature review and included two previously validated tools: the Satisfaction assessed by Employees in Health Care (SEHC)^21^ and the Short Warwick–Edinburgh Mental Wellbeing Scale (SWEMWBS).^22^ Participants were also asked if they would recommend their facility to other MOs and to give their facility a rating out of 10. The other questions were content validated in a SUFPREN workshop. All feedback was incorporated into the questionnaire and the final version was approved.

An individualised link for the questionnaire in REDCap was sent via email to the MOs and a public access link was also available. MOs were also given the option to complete a paper-based version. Family physicians identified the MOs and assisted with dissemination of the questionnaire.

### Data analysis

Data were exported from REDCap and analysed in the SPSS (version 25) software. Categorical data were reported as frequencies and percentages. Numerical data were reported as means and standard deviations if normally distributed, or medians and interquartile ranges (IQR) if not normally distributed. Participants who indicated that they planned on staying for >4 years were considered ‘retained’. Family physicians recommended this threshold as indicating a long-term commitment.

Inferential statistics was used to make inferences from the data about variables associated with retention. If the independent variable was categorical then a χ^2^ test was used, if numerical and normally distributed an independent samples *t*-test was used, and if not normally distributed a Mann–Whitney U-test. All factors associated with retention with *P*<0.1 were loaded into a multiple stepwise forward logistic regression for a multiple variable analysis.

## Results

A total of 95 MOs responded to the survey (Table 1), of whom the majority were women (54.7%), aged 30–39 years (57.9%), Afrikaans-speaking (52.6%), Christian (69.5%), in a married or long-term relationship (72.6%), and had children (52.6%).

**Table 1.** Characteristics of the participants (*n* = 95)

Variables	*n*	%
**Sex**		
Female	52	54.7
Male	43	45.3
**Age, years**		
25–29	19	20.0
30–39	55	57.9
40–49	11	11.6
50–59	5	5.3
60–65	5	5.3
**Home language**		
Afrikaans	50	52.6
English	42	44.2
Zulu	1	1.1
isiXhosa	0	0.0
Other	2	2.1
**Religion**		
Christian	66	69.5
Muslim	11	11.6
None	18	18.9
**Marital status**		
Married or long-term partner	69	72.6
Divorced or separated	6	6.3
Widowed	1	1.1
Single	19	20.0
**Partner staying in same place (*n* = 69**)		
Yes	64	92.8
No	5	7.2
**Children**		
None	45	47.4
Pre-primary school	32	33.7
Primary school	16	16.8
High school	4	4.2
Tertiary education	8	8.4

Table 2 presents the employment characteristics of the responders. Approximately half of the MOs were in the rural district (50.5%) and half in the metropolitan health services (49.5%). Approximately half were recently qualified grade 1 MOs (49.5%), and MOs had an overall median of 4 years of employment (IQR 2–8). Almost all were in full-time posts (91.6%) and the majority were in district hospitals (60.0%).

**Table 2.** Employment characteristics (*n* = 95)

Variables	*n*	%
**District**		
Cape Town Metropole	47	49.5
Cape Winelands	21	22.1
Garden Route	12	12.6
West Coast	6	6.3
Overberg	9	9.5
**Grade of medical officer**		
Grade 1	47	49.5
Grade 2	26	27.4
Grade 3	22	23.2
**Contractual commuted overtime group**		
None	21	22.1
5–12 hours	17	17.9
13–20 hours	44	46.3
>20 hours	13	13.7
**Type of employment**		
Permanent full-time	87	91.6
Permanent part-time	3	3.2
Contracted	5	5.3
**Type of facility**		
District hospital	57	60.0
Primary care	38	40.0

The majority planned to stay in the South African public sector (71.6%), although only 51.6% saw themselves staying in the DHS, and only 41.1% in the same facility (Table 3). Only 7.4% planned to specialise in family medicine, while 33.7% intended to specialise outside the DHS. In addition, 14.7% were considering alternative careers. The majority reported that they might recommend the facility to others (87.3%) and were satisfied with their experience of management, job content, and coworkers. In addition, the majority had a good score for mental wellbeing.

**Table 3.** Likelihood of retention, overall satisfaction with district health service and mental wellbeing

Variables	*n*	%
**Plans to stay in current post**		
<1 year	5	5.3
1–2 years	29	30.5
3–4 years	22	23.2
>4 years	39	41.1
Long-term career plan		
Permanent medical officer	42	44.2
Family physician	7	7.4
Hospital specialist	32	33.7
Other career	14	14.7
**5-year plan by sector**		
South African public sector	68	71.6
South African private sector	10	10.5
Emigrate	7	7.4
Other	10	10.5
**Would you recommend this facility**		
Definitely no	2	2.1
Maybe no	10	10.5
Maybe yes	42	44.2
Definitely yes	41	43.1
	**Median (IQR**)	**% satisfied**
**Overall satisfaction**		
Satisfaction with management	2.9 (2.4–3.4)	82.4
Satisfaction with job content	2.7 (2.3–3.2)	79.3
Satisfaction with coworkers	3.2 (2.7–3.8)	95.7
	**Median (IQR**)	**% score >21**
Mental wellbeing score	24.5 (20.0–28.6)	75.0

Satisfaction assessed by Employees in Health Care (SEHC) tool. Mental wellbeing assessed by Short Warwick–Edinburgh Mental Wellbeing Scale (SWEMWBS).

Supplementary Table 1 presents the association between characteristics of the responders and their employment and retention. Age was significantly associated with retention: retention median age was 37 years (95% confidence interval [CI] = 35 to 41); not retained median age was 32 years (95% CI = 32 to 34), *P* = 0.004). Sex, religion, home language, and marital status were not associated with retention. However, having a partner (*n* = 69) and not being able to live with them was significantly associated with leaving: retained 0 (0.0%), not retained five (13.2%), *P* = 0.036. There was also a significant association between the MOs having a strong local social support system and educational opportunities for their children and retention.

The type of employment and overtime contract were not associated with retention (Supplementary Table 1). The likelihood of retention increased progressively and significantly with the grade of MO. There was no association between working in rural versus metropolitan health services, or with the type of health facility, and retention. There was no association with security concerns, remuneration, or additional rural allowance and retention. Not surprisingly, those who had the intention to specialise, leave the profession, enter the private sector, or emigrate were more likely to leave.

Table 4 presents the association between satisfaction with their work experience and mental wellbeing with retention. The association between retention and satisfaction with management, job content, and coworkers did not reach statistical significance. However, the overall rating of the facility was significantly associated with retention: retention median score 8 (95% CI = 8 to 9) versus not retained median score 7 (95% CI = 7 to 8), *P* = 0.001. There was no association between the score for mental wellbeing and retention.

**Table 4.** Association between satisfaction with the work experience and mental wellbeing and retention

Variable	Retained mean score	Not retained mean score	Mean difference (95% CI)	*P* value
Satisfaction with management	3.0	2.8	0.2 (-0.4 to 0.0)	0.067
Satisfaction with job content	2.8	2.7	0.1 (-0.3 to 0.0)	0.084
Satisfaction with coworkers	3.4	3.2	0.2 (-0.4 to 0.0)	0.068
Mental wellbeing score	25.4	23.9	1.5 (-3.4 to 0.4)	0.124

The multiple variable analysis only retained two factors in the model. These were overall rating of the facility (adjusted OR [AOR] 1.4 [95% CI = 1.0 to 1.9], *P* = 0.04) and the MO having their partner stay with them (AOR 2.8 [95% CI = 1.0 to 8.1], *P* = 0.05).

## Discussion

### Summary

Most MOs planned to stay in the public sector (71.6%), although only 51.6% in the DHS and only 41.1% at the same facility. The overall rating of the facility was one of the key factors in determining retention. It was difficult to evaluate what issues were important in this rating as satisfaction with management, job content, and coworkers were not significantly associated with retention. Interestingly, none of the expected factors related to employment were significant (such as remuneration, overtime, safety, type of facility, rural or metropolitan location, or type of contract). Factors related to social and family life, however, were important, and being able to live with one’s partner was significantly associated with retention. Not surprisingly, retention was linked to the grade of MO and career intentions. More senior MOs who had already stayed in the DHS were more likely to continue to do so. Those who planned to specialise, emigrate, enter the private sector, or change professions were also more likely to leave.

### Strengths and limitations

The survey achieved a 76.0% response rate, which is close to the desired 80% and supports the reliability of these findings. Facilities were included from all districts and with an almost equal rural and urban distribution of MOs. The survey pointed towards useful issues that could be explored further in the qualitative study that came afterwards.^20^

The selection of facilities was related to the family physicians’ participation in the SUFPREN and although the response rate was acceptable, the total sample was still relatively small at 95 MOs. Facilities without family physicians were excluded and were often smaller and more remote district hospitals. These findings from the Western Cape cannot be statistically generalised to other provinces, but the issues identified are likely to be relevant.

### Comparison with existing literature

Following this survey, a descriptive exploratory qualitative study attempted to unpack what factors were influencing the rating of the facility.^20^ This study suggested that teamwork, support, and supervision were critical. Being part of a cohesive and caring clinical team was important, as well as having access to support and supervision when needed from senior colleagues. Other key factors were having a collaborative, responsive, and appreciative management, being part of a rich learning environment, dealing with a manageable workload, and having a family physician.

The present survey also pointed towards personal and family issues, and the qualitative study highlighted the importance of being with one’s partner, prioritising parenting and childcare, and being close to social support such as family and friends.^20^ Management needed to be flexible and sensitive to contractual and overtime issues that might impact on parenting and childcare needs.

A number of factors noted in the literature^11–16^ were not important in this context. For example, MOs were largely satisfied with their remuneration, infrastructure, resources, basic living, and working conditions. In many other African countries, this is not the case.^15,23^ Interestingly, there was no distinction between retention in rural and urban areas or between district hospitals and PHC. Rural areas of the Western Cape may be less remote and impoverished when compared with other provinces and other African countries.

Only a small proportion of MOs were considering a career in family medicine, which would retain them in the DHS while offering them seniority and career advancement. This was also noted in the qualitative study, where MOs were positive about family physicians, but did not want to become one.^20^ This reluctance was linked to concerns about having to develop expertise across all clinical areas, the scarcity of family physician posts on qualifying, the difficulty of obtaining a registrar post in rural areas, and the possibility that they would have to relocate in order to train. A recent national position paper recommends a goal of one family physician for each district hospital, community health centre, or sub-district by 2030 and notes that an increase in both registrar and family physician posts will be needed to achieve this goal.^24^

### Implications for practice

In many ways the Western Cape appears to be doing well in creating a solid foundation to retain MOs. It is clear, however, that the overall rating of the facility is still a key factor that can be influenced by the health services and a number of issues may be important:^20^

- Being part of a cohesive, supportive and caring clinical team
- Having support and supervision, including from a family physician
- Shifting to a more approachable, collaborative, and supportive management style
- Ensuring a learning environment that enables ongoing development
- Keeping the workload manageable
- Flexibility in terms of overtime and contracts to allow parenting and childcare

These issues are likely to apply across the South African context and be important for both district and facility management teams. Strengthening DHS remains a priority for the government, especially with the intention to introduce national health insurance.

## Data

The dataset relied on in this article is available from the corresponding author on reasonable request.

## References

[1] World Health Organization (WHO), United Nations Children’s Fund (‎‎UNICEF)‎‎ (2020). Operational framework for primary health care: transforming vision into action.

[2] WHO (2018). Astana declaration on primary health care. https://www.who.int/primary-health/conference-phc/declaration.

[3] WHO, UNICEF (2022). Primary health care measurement framework and indicators: monitoring health systems through a primary health care lens. https://www.who.int/publications/i/item/9789240044210.

[4] National Department of Health (2020). 2030 Human resources for health strategy: investing in the health workforce for universal health coverage.

[5] National Department of Health Ideal Clinic South Africa. https://www.idealhealthfacility.org.za.

[6] De Villiers M, De Villiers P (2006). The knowledge and skills gap of medical practitioners delivering district hospital services in the Western Cape, South Africa. S Afr Fam Pract.

[7] Erumeda NJ, Couper ID, Thomas LS (2019). A self-assessment study of procedural skills of doctors in peri-urban district hospitals of Gauteng, South Africa. Afr J Prim Health Care Fam Med.

[8] Wilson NW, Couper ID, De Vries E (2009). A critical review of interventions to redress the inequitable distribution of healthcare professionals to rural and remote areas. Rural Remote Health.

[9] Tiwari R, Mash R, Karangwa I, Chikte U (2021). A human resources for health analysis of registered family medicine specialists in South Africa: 2002–19. Fam Pract.

[10] Mash R, Malan Z, von Pressentin K, Blitz J (2015). Strengthening primary health care through primary care doctors: the design of a new national Postgraduate Diploma in Family Medicine. S Afr Fam Pract.

[11] Mbemba GIC, Gagnon M-P, Hamelin-Brabant L (2016). Factors influencing recruitment and retention of healthcare workers in rural and remote areas in developed and developing countries: an overview. J Public Health Afr.

[12] Lehmann U, Dieleman M, Martineau T (2008). Staffing remote rural areas in middle- and low-income countries: a literature review of attraction and retention. BMC Health Serv Res.

[13] WHO (2021). WHO guideline on health workforce development, attraction, recruitment and retention in rural and remote areas. https://www.who.int/publications/i/item/9789240024229.

[14] Willis-Shattuck M, Bidwell P, Thomas S (2008). Motivation and retention of health workers in developing countries: a systematic review. BMC Health Serv Res.

[15] Bonenberger M, Aikins M, Akweongo P, Wyss K (2014). The effects of health worker motivation and job satisfaction on turnover intention in Ghana: a cross-sectional study. Hum Resour Health.

[16] Marchand C, Peckham S (2017). Addressing the crisis of GP recruitment and retention: a systematic review. Br J Gen Pract.

[17] Western Cape Government: Health (2021). Annual report 2020–21. https://www.westerncape.gov.za/assets/annual_report_2020_2021_0.pdf.

[18] Government of South Africa (2013). National Development Plan Annual report 2020-21. Cape Town.

[19] Mash R (2020). Establishing family physician research networks in South Africa. S Afr Fam Pract (2004).

[20] Mash RJ, Viljoen W, Swartz S (2022). Retention of medical officers in the district health services of the Western Cape, South Africa: an exploratory descriptive qualitative study. S Afr Fam Pract (2004).

[21] Alpern R, Canavan ME, Thompson JT (2013). Development of a brief instrument for assessing healthcare employee satisfaction in a low-income setting. PLoS One.

[22] Murray MA, Cardwell C, Donnelly M (2017). GPs’ mental wellbeing and psychological resources: a cross-sectional survey. Br J Gen Pract.

[23] Nkomazana O, Mash R, Shaibu S, Phaladze N (2015). Stakeholders ’ perceptions on shortage of healthcare workers in primary healthcare in Botswana: focus group discussions. PLoS One.

[24] South African Academy of Family Physicians (2022). The contribution of family physicians to district health services in South Africa: a national position paper by the South African Academy of family physicians. S Afr Fam Pract (2004).

